# 1412. Clinical Epidemiology and Characteristics of Pulmonary Nontuberculous Mycobacterial Isolates from a Large Academic Military Treatment Facility

**DOI:** 10.1093/ofid/ofab466.1604

**Published:** 2021-12-04

**Authors:** Mary B Ford, Jason Okulicz, Jesse Salinas, John Kiley

**Affiliations:** 1 Brooke Army Medical Center, San Antonio, Texas; 2 Brooke Army Medical Center, JBSA Fort Sam Houston, TX, San Antonio, Texas

## Abstract

**Background:**

Non-tuberculous mycobacteria (NTM) are ubiquitous in the environment and include pathogenic and nonpathogenic species. Although prevalence appears to be increasing in the US, diagnosis and treatment can be challenging. This study describes the epidemiology and clinical characteristics of pulmonary NTM (pNTM) isolates at Brooke Army Medical Center (BAMC).

**Methods:**

BAMC pulmonary NTM isolates from 2012-2020 were included. Corresponding electronic health records were reviewed for epidemiologic, microbiologic, and clinical data. Pulmonary NTM infection (pNTMi) was defined using 2020 NTM guidelines and patients were divided into 2 groups based on whether guideline criteria for pNTMi were met. Demographic, microbiologic, and clinical characteristics were compared between groups.

**Results:**

A total of 813 isolates from 225 patients were analyzed (median 2 [IQR 1-4] isolates per patient). Approximately half (49.7%) were female with a median age of 71 years (IQR 62-79, Table 1), and the majority were current or former smokers (57.3%). Compared to those not meeting criteria (n=116; 51.6%), pNTMi patients (n=109; 48.4%) more commonly had bronchiectasis (47.7% vs 27.6; p=0.002) but were less likely to have solid organ malignancy (11.9% vs 23.3%; p=0.036). A higher proportion of pNTMi patients were female (58% vs 42%; p=0.005) and had lower median Body Mass Index (BMI, 22.6 vs 25.1; p=0.001). *M. avium* complex (MAC) was more common among pNTMi patients (75.2% vs 35.3%; p=0.001). In contrast, *M. simiae* and *M. gordonae* were more likely to be isolated from those not meeting criteria (25.9% vs. 10.1%; p=0.003 and 16.4% vs. 1.8%; p=0.001, respectively). Among pNTMi patients, 60 (55%) were offered therapy and were more likely to be younger (70 [IQR 63-76] vs. 73 [IQR 65-82] years; p=0.049), have chronic obstructive pulmonary disease (COPD; 51.7% vs 24.5%; p=0.006) and MAC (88.3% vs. 59.2%; p=0.001) compared to untreated patients (Table 2).

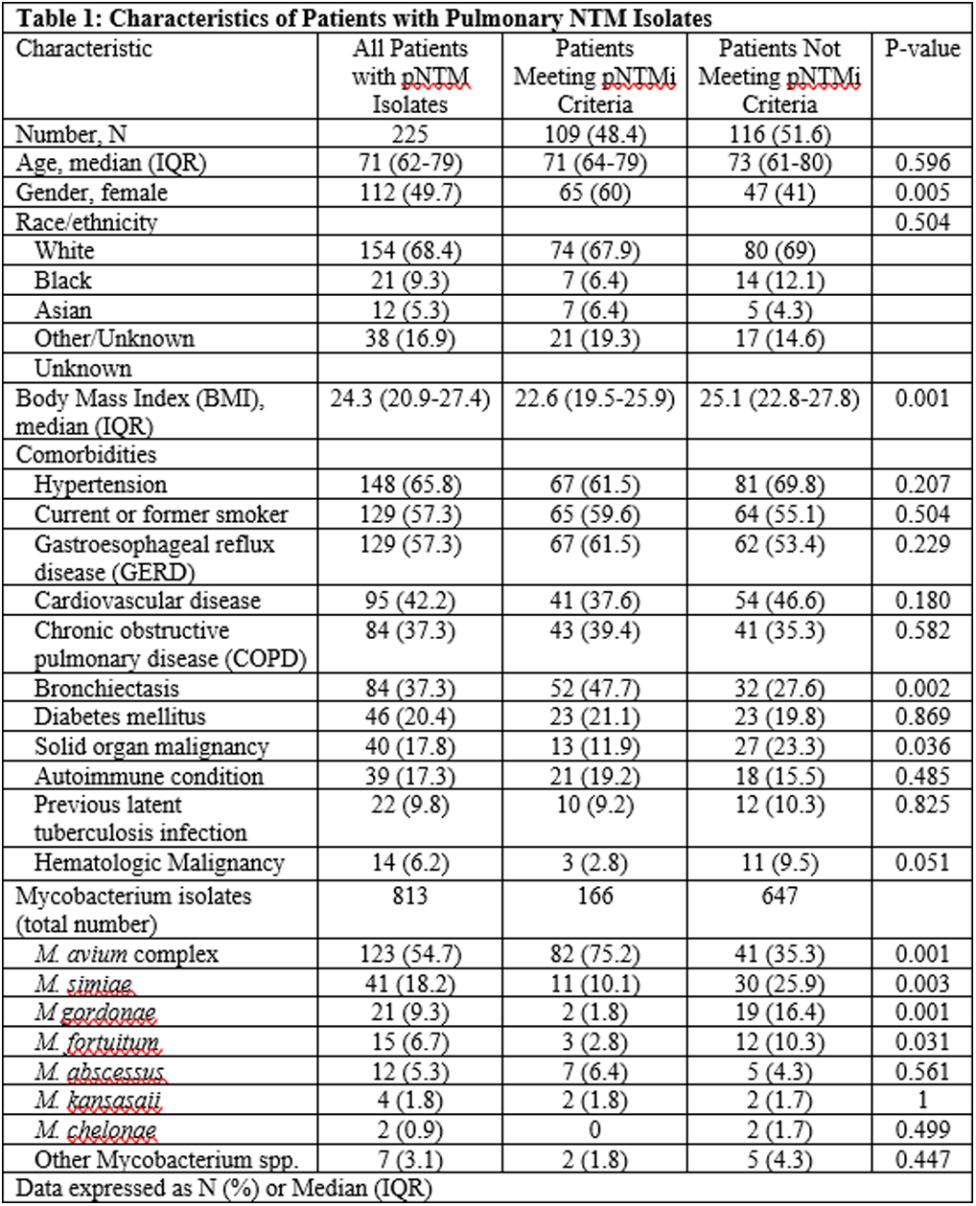

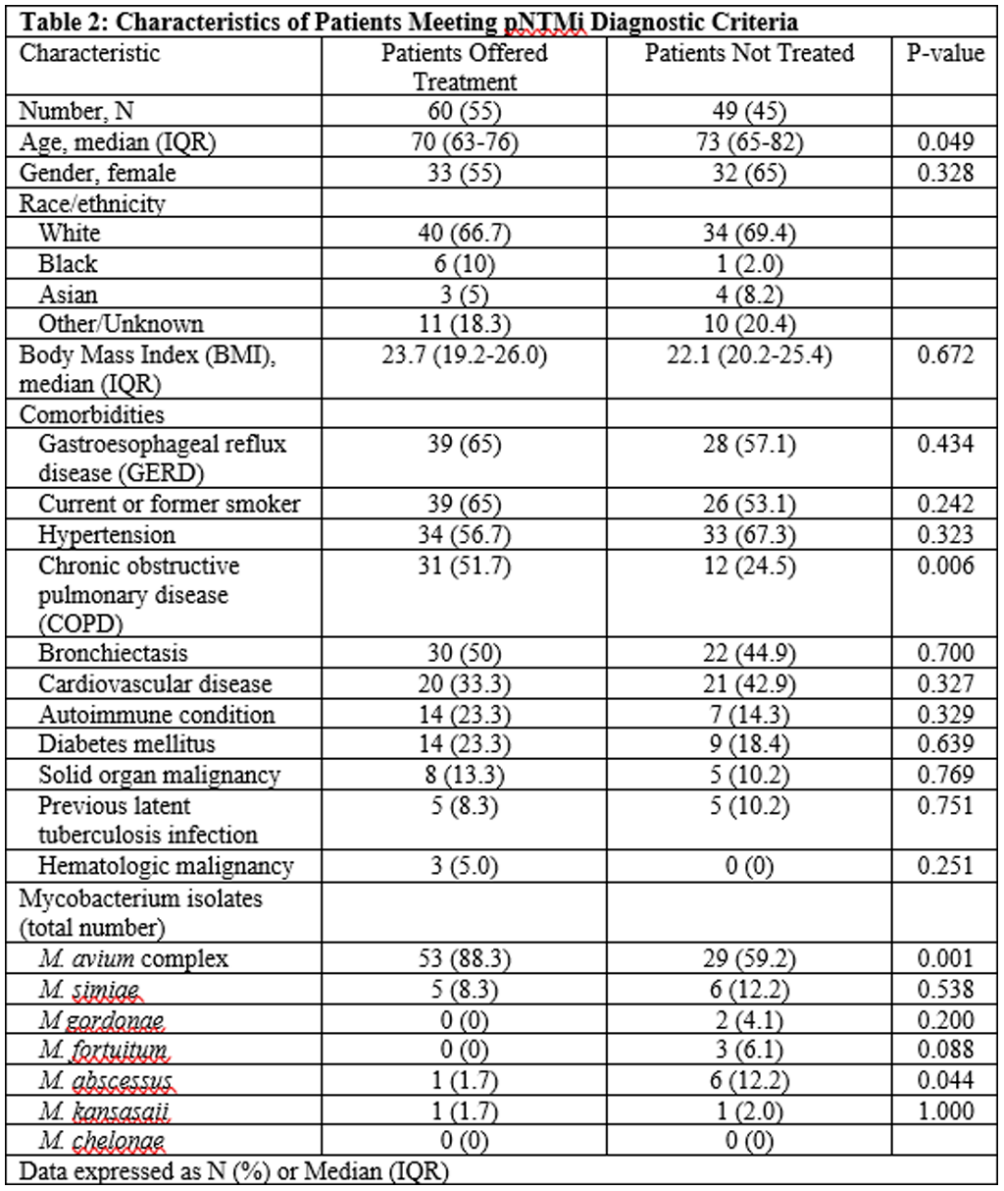

**Conclusion:**

Approximately half of pNTM isolates were observed in patients who did not meet criteria for pNTMi diagnosis. Female patients, lower BMI, bronchiectasis, or MAC isolation were more likely to meet pNTMi criteria. Management of pNTMi remains a challenge, with younger patients with COPD and MAC more likely to receive treatment.

**Disclosures:**

**All Authors**: No reported disclosures

